# Genetic landscape of congenital insensitivity to pain and hereditary sensory and autonomic neuropathies

**DOI:** 10.1093/brain/awad328

**Published:** 2023-09-28

**Authors:** Annette Lischka, Katja Eggermann, Christopher J Record, Maike F Dohrn, Petra Laššuthová, Florian Kraft, Matthias Begemann, Daniela Dey, Thomas Eggermann, Danique Beijer, Jana Šoukalová, Matilde Laura, Alexander M Rossor, Radim Mazanec, Jonas Van Lent, Pedro J Tomaselli, Martin Ungelenk, Karlien Y Debus, Shawna M E Feely, Dieter Gläser, Sujatha Jagadeesh, Madelena Martin, Geeta M Govindaraj, Pratibha Singhi, Revanth Baineni, Niranjan Biswal, Marisol Ibarra-Ramírez, Maryse Bonduelle, Burkhard Gess, Juan Romero Sánchez, Renu Suthar, Vrajesh Udani, Atchayaram Nalini, Gopikrishnan Unnikrishnan, Wilson Marques, Sandra Mercier, Vincent Procaccio, Céline Bris, Beena Suresh, Vaishnavi Reddy, Mariola Skorupinska, Nathalie Bonello-Palot, Fanny Mochel, Georg Dahl, Karthika Sasidharan, Fiji M Devassikutty, Sheela Nampoothiri, Maria J Rodovalho Doriqui, Wolfgang Müller-Felber, Katharina Vill, Tobias B Haack, Andreas Dufke, Michael Abele, Rolf Stucka, Saima Siddiqi, Noor Ullah, Stephanie Spranger, Deborah Chiabrando, Behiye S Bolgül, Yesim Parman, Pavel Seeman, Angelika Lampert, Jörg B Schulz, John N Wood, James J Cox, Michaela Auer-Grumbach, Vincent Timmerman, Jonathan de Winter, Andreas C Themistocleous, Michael Shy, David L Bennett, Jonathan Baets, Christian A Hübner, Enrico Leipold, Stephan Züchner, Miriam Elbracht, Arman Çakar, Jan Senderek, Thorsten Hornemann, C Geoffrey Woods, Mary M Reilly, Ingo Kurth

**Affiliations:** Institute for Human Genetics and Genomic Medicine, Medical Faculty, RWTH Aachen University Hospital, 52074 Aachen, Germany; Institute for Human Genetics and Genomic Medicine, Medical Faculty, RWTH Aachen University Hospital, 52074 Aachen, Germany; Department of Neuromuscular Diseases, UCL Queen Square Institute of Neurology, London WC1N 3BG, UK; Department of Neurology, Medical Faculty of the RWTH Aachen University, 52074 Aachen, Germany; Dr. John T. Macdonald Foundation, Department of Human Genetics and John P. Hussman Institute for Human Genomics, University of Miami, Miller School of Medicine, Miami, FL 33136, USA; Department of Paediatric Neurology, 2nd Faculty of Medicine, Charles University in Prague and Motol University Hospital, 150 06 Praha, Czechia; Institute for Human Genetics and Genomic Medicine, Medical Faculty, RWTH Aachen University Hospital, 52074 Aachen, Germany; Institute for Human Genetics and Genomic Medicine, Medical Faculty, RWTH Aachen University Hospital, 52074 Aachen, Germany; Institute for Human Genetics and Genomic Medicine, Medical Faculty, RWTH Aachen University Hospital, 52074 Aachen, Germany; Institute for Human Genetics and Genomic Medicine, Medical Faculty, RWTH Aachen University Hospital, 52074 Aachen, Germany; Dr. John T. Macdonald Foundation, Department of Human Genetics and John P. Hussman Institute for Human Genomics, University of Miami, Miller School of Medicine, Miami, FL 33136, USA; Department of Medical Genetics, University Hospital Brno, 625 00 Brno, Czechia; Department of Neuromuscular Diseases, UCL Queen Square Institute of Neurology, London WC1N 3BG, UK; Department of Neuromuscular Diseases, UCL Queen Square Institute of Neurology, London WC1N 3BG, UK; Department of Neurology, Faculty of Medicine, Charles University in Prague and Motol University Hospital, 150 06 Prague, Czechia; Peripheral Neuropathy Research Group, Department of Biomedical Sciences, Institute Born Bunge, University of Antwerp, 2160 Antwerp, Belgium; Department of Neurosciences and Behaviour Sciences, Clinical Hospital of Ribeirão Preto, University of São Paulo, Ribeirão Preto, 14015-130, Brazil; Institute of Human Genetics, University Hospital Jena, 07747 Jena, Germany; Center for Molecular Biomedicine Institute for Biophysics, Friedrich-Schiller Universität Jena, 07745 Jena, Germany; Department of Neurology, University of Iowa Carver College of Medicine, Iowa City, IA 52242, USA; Division of Pediatric Neurology, Seattle Children’s Hospital, University of Washington School of Medicine, Seattle, WA 98105, USA; Center for Human Genetics, Genetikum®, 89231 Neu-Ulm, Germany; Department of Clinical Genetics and Genetic Counselling, Mediscan Systems, Chennai 600032, Tamilnadu, India; Davis and Davis Children's Hospital, University of California, Sacramento, CA 95817, USA; Department of Pediatrics, Government Medical College, Kozhikode, Kerala 673 008, India; Pediatric Neurology and Neurodevelopment, Medanta, The Medicity, Gurgaon, Haryana 122 001, India; Department of Pediatrics, Jawaharlal Institute of Postgraduate Medical Education and Research, Puducherry 605 006, India; Department of Pediatrics, Jawaharlal Institute of Postgraduate Medical Education and Research, Puducherry 605 006, India; Genetics Department, Hospital Universitario Dr. José Eleuterio González Universidad Autónoma de Nuevo León, 64460 Monterrey, Nuevo León, México; Centre for Medical Genetics, Universitair Ziekenhuis Brussel, 1090 Jette, Brussels, Belgium; Department of Neurology, Medical Faculty of the RWTH Aachen University, 52074 Aachen, Germany; Department of Neurology, University Hospital, Evangelisches Klinikum Bethel, University of Bielefeld, 33617 Bielefeld, Germany; Pediatría, Clínica Premium, 29601 Marbella, Spain; Pediatric Neurology and Neurodevelopment Unit, Department of Pediatrics, Advanced Pediatric Centre, Post Graduate Institute of Medical Education and Research (PGIMER), Chandigarh 160 012, India; Department of Child Neurology, PD Hinduja Hospital and Medical Research Centre, Mumbai, Maharashtra 400 016, India; Department of Neurology, National Institute of Mental Health and Neurosciences, Bengaluru 560 029, India; Department of Neurology, National Institute of Mental Health and Neurosciences, Bengaluru 560 029, India; Department of Neurosciences and Behaviour Sciences, Clinical Hospital of Ribeirão Preto, University of São Paulo, Ribeirão Preto, 14015-130, Brazil; CHU Nantes, Service de Génétique Médicale, Centre de Référence des Maladies Neuromusculaires AOC, 44000 Nantes, France; Department of Biochemistry and Genetics, MitoVasc Institute, UMR CNRS 6015- INSERM U1083, CHU Angers, 49055 Angers, France; Department of Biochemistry and Genetics, MitoVasc Institute, UMR CNRS 6015- INSERM U1083, CHU Angers, 49055 Angers, France; Department of Clinical Genetics and Genetic Counselling, Mediscan Systems, Chennai 600032, Tamilnadu, India; Department of Clinical Genetics and Genetic Counselling, Mediscan Systems, Chennai 600032, Tamilnadu, India; Department of Neuromuscular Diseases, UCL Queen Square Institute of Neurology, London WC1N 3BG, UK; INSERM, MMG, U 1251, Marseille, France, Aix Marseille Univ, 13385 Marseille, France; Genetics Department, Sorbonne Université, Paris Brain Institute, APHP, INSERM, CNRS, 75013 Paris, France; Pediatric Neurology, Children’s Hospital of the King’s Daughters in Norfolk, Norfolk, VA 23507, USA; Department of Pediatrics, Government Medical College, Kozhikode, Kerala 673 008, India; Department of Pediatrics, Government Medical College, Kozhikode, Kerala 673 008, India; Department of Pediatric Genetics, Amrita Institute of Medical Sciences and Research Center, Cochin, Kerala 682 041, India; Department of Genetics, Hospital Infantil Doutor Juvêncio Mattos, São Luis, Maranhão 65015-460, Brazil; Department of Neuropediatrics, Developmental Neurology and Social Pediatrics, LMU Campus Innenstadt, University of Munich, 80337 Munich, Germany; Department of Pediatric Neurology and Developmental Medicine, Dr. von Hauner Children's Hospital, University Hospital, LMU Munich, 80337 Munich, Germany; Institute of Human Genetics, School of Medicine, Technical University of Munich, 81675 Munich, Germany; Institute of Medical Genetics and Applied Genomics, University of Tübingen, 72076 Tübingen, Germany; Institute of Medical Genetics and Applied Genomics, University of Tübingen, 72076 Tübingen, Germany; Neurologie, Praxis für Neurologie und Schlafmedizin, 53359 Rheinbach, Germany; Friedrich Baur Institute at the Department of Neurology, LMU University Hospital, LMU Munich, 80336 Munich, Germany; Genomics Group, Institute of Biomedical and Genetic Engineering (IBGE), Islamabad 44000, Pakistan; Institute for Paramedical Sciences, Khyber Medical University, Peshawar, KPK 25100, Pakistan; MVZ Humangenetik Bremen, Limbach Genetics, 28209 Bremen, Germany; Department of Molecular Biotechnology and Health Sciences, Molecular Biotechnology Center ‘Guido Tarone’, University of Torino, 10124 Turin, Italy; Department of Pedodontics, Faculty of Dentistry, Dicle University, 21200 Diyarbakir, Turkey; Neuromuscular Unit, Department of Neurology, Istanbul Faculty of Medicine, Istanbul University, 34093 Istanbul, Turkey; Department of Paediatric Neurology, 2nd Faculty of Medicine, Charles University in Prague and Motol University Hospital, 150 06 Praha, Czechia; Institute of Neurophysiology, Medical Faculty, Uniklinik RWTH Aachen University, 52074 Aachen, Germany; Department of Neurology, Medical Faculty of the RWTH Aachen University, 52074 Aachen, Germany; JARA-BRAIN Institute Molecular Neuroscience and Neuroimaging, Research Centre Jülich GmbH, and RWTH Aachen University, 52056 Aachen, Germany; Molecular Nociception Group, Wolfson Institute for Biomedical Research, University College London, London WC1E 6BT, UK; Molecular Nociception Group, Wolfson Institute for Biomedical Research, University College London, London WC1E 6BT, UK; Department of Orthopedics and Trauma Surgery, Medical University of Vienna, 1090 Vienna, Austria; Peripheral Neuropathy Research Group, Department of Biomedical Sciences, Institute Born Bunge, University of Antwerp, 2160 Antwerp, Belgium; Translational Neurosciences and Institute Born Bunge, Faculty of Medicine and Health Sciences, University of Antwerp, 2610 Antwerp, Belgium; Neuromuscular Reference Centre, Department of Neurology, Antwerp University Hospital, 2610 Antwerp, Belgium; Nuffield Department of Clinical Neuroscience, University of Oxford, Oxford OX3 9DU, UK; Department of Neurology, University of Iowa Carver College of Medicine, Iowa City, IA 52242, USA; Nuffield Department of Clinical Neuroscience, University of Oxford, Oxford OX3 9DU, UK; Translational Neurosciences and Institute Born Bunge, Faculty of Medicine and Health Sciences, University of Antwerp, 2610 Antwerp, Belgium; Neuromuscular Reference Centre, Department of Neurology, Antwerp University Hospital, 2610 Antwerp, Belgium; Institute of Human Genetics, University Hospital Jena, 07747 Jena, Germany; Department of Anesthesiology and Intensive Care and CBBM—Center of Brain, Behavior and Metabolism, University of Luebeck, 23562 Luebeck, Germany; Dr. John T. Macdonald Foundation, Department of Human Genetics and John P. Hussman Institute for Human Genomics, University of Miami, Miller School of Medicine, Miami, FL 33136, USA; Institute for Human Genetics and Genomic Medicine, Medical Faculty, RWTH Aachen University Hospital, 52074 Aachen, Germany; Neuromuscular Unit, Department of Neurology, Istanbul Faculty of Medicine, Istanbul University, 34093 Istanbul, Turkey; Friedrich Baur Institute at the Department of Neurology, LMU University Hospital, LMU Munich, 80336 Munich, Germany; Department of Clinical Chemistry, University Hospital Zurich, University of Zurich, 8006 Zurich, Switzerland; Cambridge Institute for Medical Research, Keith Peters Building, Cambridge Biomedical Campus, Cambridge CB2 0XY, UK; Department of Neuromuscular Diseases, UCL Queen Square Institute of Neurology, London WC1N 3BG, UK; Institute for Human Genetics and Genomic Medicine, Medical Faculty, RWTH Aachen University Hospital, 52074 Aachen, Germany

**Keywords:** neuropathies, CIP, HSAN, HSN, pain, genetics

## Abstract

Congenital insensitivity to pain (CIP) and hereditary sensory and autonomic neuropathies (HSAN) are clinically and genetically heterogeneous disorders exclusively or predominantly affecting the sensory and autonomic neurons. Due to the rarity of the diseases and findings based mainly on single case reports or small case series, knowledge about these disorders is limited.

Here, we describe the molecular workup of a large international cohort of CIP/HSAN patients including patients from normally under-represented countries. We identify 80 previously unreported pathogenic or likely pathogenic variants in a total of 73 families in the >20 known CIP/HSAN-associated genes. The data expand the spectrum of disease-relevant alterations in CIP/HSAN, including novel variants in previously rarely recognized entities such as *ATL3*-, *FLVCR1*- and *NGF*-associated neuropathies and previously under-recognized mutation types such as larger deletions. *In silico* predictions, heterologous expression studies, segregation analyses and metabolic tests helped to overcome limitations of current variant classification schemes that often fail to categorize a variant as disease-related or benign.

The study sheds light on the genetic causes and disease-relevant changes within individual genes in CIP/HSAN. This is becoming increasingly important with emerging clinical trials investigating subtype or gene-specific treatment strategies.

## Teaser

Lischka *et al.* describe the molecular workup of a large cohort of patients with congenital insensitivity to pain/hereditary sensory and autonomic neuropathies. They identify 80 previously unreported pathogenic or likely pathogenic variants in 73 families, broadening the known mutational spectrum of these rare conditions.

## Introduction

Complex genetic variability leads to individual differences in the perception of pain. In contrast to polygenic and environmental correlations, specific single nucleotide variants can have an effect such that the sensation of pain is absent from birth, or a progressive loss of pain sensitivity becomes apparent in the course of life. In these rare and monogenic diseases, there is usually a developmental disorder of pain-sensing neurons, neurodegeneration of peripheral nerves, or altered electrical activity of nociceptors. This heterogeneous group of genetic pain loss disorders includes congenital insensitivity to pain (CIP), hereditary sensory neuropathy (HSN) and, if autonomic nerves are involved, hereditary sensory and autonomic neuropathy (HSAN). The HSNs are assigned here to the group of HSAN diseases. The consequences of pain loss are recurrent injuries and fractures resulting in mutilation or amputation, often in combination with severely impaired wound healing. The sensation of itch, temperature and touch may also be impaired with negative impact on health. Affected patients can have marked autonomic dysfunction, such as anhidrosis, gastrointestinal and sexual dysfunction or blood pressure fluctuations. In some subtypes of CIP/HSAN, patients also show intellectual disability, muscle weakness, ataxia or other additional symptoms. To date, pathogenic variants in more than 20 genes are known to cause pain loss syndromes. Various cellular processes can be affected, including sodium channel activity,^1,2^ sphingolipid metabolism,^3,4^ membrane dynamics,^5–7^ axonal transport,^8,9^ neurotrophin signalling,^10,11^ epigenetic regulation^12^ or cytoskeletal architecture.^13^ Because of the rarity of CIP/HSAN disorders, knowledge of these conditions is limited and diagnosis is often delayed or incorrect resulting in a diagnostic odyssey. Moreover, except for few studies including larger patient numbers,^14,15^ the literature is often restricted to single case descriptions. Detailed studies on the genetic spectrum of the respective molecular subtypes have been largely lacking to date, but as the first therapeutic approaches for certain subtypes are in clinical trials, molecular classification is becoming increasingly important.^16–18^

In this retrospective study, we provide deeper insights into the clinical and genetic landscape of these rare conditions by sequencing of the as yet largest cohort of CIP/HSAN patients.

## Materials and methods

### Patient cohort

Genetic data were collected retrospectively from existing datasets from patients who had been either referred directly to the participating centres or whose blood samples and clinical information were sent to the participating centres. All patients showed clinical signs of HSAN or CIP (i.e. reduced sensation of pain, temperature and touch either congenital or developed later in life and/or clinical manifestations such as unnoticed injuries, skin ulcerations, amputations, osteomyelitis, painless fractures). Since neuropathic pain, especially in the initial stage, may be a sign of different forms of HSAN, patients with a combined phenotype and a variant in one of the HSAN-related genes were also included in the study. Patients with suspected other genetic disorders potentially mimicking the phenotype of HSAN, such as Charcot-Marie-Tooth disease (CMT) or e.g. Lesch-Nyhan syndrome as a typical example with self-mutilating behaviour, were not included in the study. Other possible underlying non-genetic causes of decreased pain sensitivity (e.g. toxic, metabolic or infectious causes of polyneuropathy) were queried by the respective clinical centres and resulted in exclusion from the study. The study was conducted in accordance with the Declaration of Helsinki and has been approved by the local ethics committees of the participating institutions. Prior to inclusion, written informed consent was obtained from patients or their legal guardians [Ethics approval Uniklinik RWTH Aachen: EK 086-20; Ethics approval London: 09/H0716/61 (‘CMT—A natural history study’); Ethics approvals University of Oxford: 12/LO/0017 (Painful Channelopathies Study, https://clinicaltrials.gov/ct2/show/NCT02696746), 18/SC/0263 (Pain in Peripheral Nerve Lesions), 13/EE/0325 (NIHR BioResource—Rare Diseases; Ethics approval of Antwerp and University Hospital of Antwerp: B300201422160 (V.T.) and B300201525715 (J.B.); Ethics approval University Hospital of Tübingen: 116/2015BO2].

### Short-read next-generation sequencing

Project sites and collaborating research institutions provided genetic data of CIP/HSAN patients that had been analysed by short-read sequencing. Datasets were analysed with regard to novel variants in known CIP/HSAN genes [genes from the panel ‘pain syndromes’ v1.12, Genomics England Panel App were prioritized (https://panelapp.genomicsengland.co.uk/panels/288/)]. Variant calling was done by each research institute separately; protocols, consumables and pipelines used differed between the institutions. Detailed protocols can be provided upon request. Cases were considered if mono- or biallelic variants (for dominant and recessive disorders, respectively) were found in one of the core genes with no additional probably disease-associated variants detected by screening of the datasets. The MasterMind database was checked in June 2023 and variants were included if they (i) had not been published in the literature at all; (ii) had only been described in supplementary materials; or (iii) had only been reported in patients with a phenotype other than CIP/HSAN (Supplementary Table 1). If possible, segregation analyses within the families were performed using Sanger sequencing.

### Modified classification of pathogenicity

In accordance with the American College of Medical Genetics (ACMG) criteria,^19^ in a first step, only pathogenic or likely pathogenic variants were selected consistent with a very high probability of molecular diagnostic confirmation (Table 1). Subsequently, variants with formally unclear clinical significance (VUS) in the core genes were reassessed (Table 2). For this purpose, five additional objective criteria were established to cover variant features that support the pathogenicity of a variant but are not represented in the actual ACMG guidelines so far. Two novel criteria were classified as moderate and three as supporting (Supplementary Table 2). Additionally, we established the term of VUS+, defined as variants that do not meet the original criteria for likely pathogenic or pathogenic variants but fulfil at least one of the new criteria supporting their pathogenicity.

**Table 1:** Novel (likely) pathogenic variants in CIP/HSAN genes

Patient	Novel variant	Genotype	ACMG	Inh.	PP	Au	Mo	SL	F/M	ID
***DST* (NM_001374736)**										
4	c.4849C>T, p.(Arg1617*)	het + c.19942G>A (het)	LPV	AR	red		−	+	+	−
5	c.22513C>T, p.(Arg7505*)	comp + c.19451A>T	LPV	AR	red	+	+	+	+	−
***FLVCR1* (NM_014053)**										
6	c.139_151del, p.(Phe47Glyfs*62)	het + c.722C>T (het)	LPV	AR	red	−	+	−	+	+
7	c.868_871del, p.(Ile290*)	comp + c.655G>A	LPV	AR	abs		+	−	−	−
10	c.1318_1321del, p.(Thr440Valfs*63)	comp + c.1317G>A	LPV	AR	red		+			−
11	c.1194C>A, p.(Tyr398*)	comp + c.1526-3C>T	LPV	AR	abs		−	+	+	−
***KIF1A* (NM_001244008)**										
12	c.2839dup, p.(Leu947Profs*49)	hom°	LPV	AR	red		+	−	−	−
***NGF* (NM_002506)**										
13	c.524_525del, p.(Phe175*)	hom°	LPV	AR	red		+		+	+
14	c.695_696del, p.(Val232Alafs*39)	hom°	LPV	AR		+		+	+	+
***NTRK1* (NM_002529)**										
15	c.2T>A, p.(Met1?)	hom	LPV	AR	*					
16	c.145C>T, p.(Arg49*)	hom°	LPV	AR	abs	+	−	+	+	
17	c.213-1G>A, p.?	hom°	LPV	AR	abs	+				
18	c.228_229delGCinsTT, p.(Gln76_Gln77delinsHis*)	hom	LPV	AR	*					
19	c.287+2T>A, p.?	comp + known LPV	LPV	AR	*					
22, 23	c.605del, p.(Asn202Metfs*37)	het + known PV (het)	LPV	AR	red	+	−	+	+	+
24	c.717+1del, p.?	hom	LPV	AR	*					
27	c.850del, p.(Phe284Serfs*186)	hom	LPV	AR	*					
28	c.851-2A>G, p.?	hom°	LPV	AR	*					
30	c.1320del, p.(Asn440Lysfs*30)	hom	LPV	AR	abs	+				
32, 33	c.1865del, p.(Leu622Argfs*36)	hom°	LPV	AR	red	+	−	+	+	−
34	c.1953_1954insT, p.(Ala652Cysfs*17)	hom	LPV	AR	*					
***PRDM12* (NM_021619)**										
37	c.575T>A, p.(Ile192Asn)	hom°	LPV	AR	abs	−	−	+	+	
38	c.788G>A, p.(Arg263His)	comp + known LPV	LPV	AR	abs	+				−
***SCN9A* (NM_002977)**										
41	c.116del, p.(Lys39Argfs*51)	hom	LPV	AR	*					
42	c.515T>G, p.(Leu172Arg)	hom°	LPV	AR	abs	+	−		+	−
43	c.793C>T, p.(Gln265*)	hom	LPV	AR	*					
44	c.809_822del, p.(Asn270Metfs*7)	comp + c.1927C>T	LPV	AR	*					
45	c.954_955del, p.(Thr319Argfs*19)	hom	LPV	AR	*					
46	c.1368del, p.(Gly457Alafs*12)	hom	LPV	AR	*					
47	c.1449del, p.(Asn484Ilefs*81)	comp + known PV	PV	AR	*					
48	c.1602+2del, p.?	comp + known LPV	LPV	AR	abs					
44	c.1927C>T, p.(Gln643*)	comp + c.809_822del	LPV	AR	*					
50	c.2109G>A, p.(Trp703*)	comp + known PV	PV	AR	*					
51, 52	c.2362dup, p.(Asp788Glyfs*4)	hom°	LPV	AR	red	−	−	+	+	−
54	c.3309del, p.(Tyr1103*)	comp + c.5340del	LPV	AR	abs	−	−		+	−
55	c.4331del, p.(Val1444Alafs*3)	hom	LPV	AR	red	−	−	+	−	−
56	c.4467del, p.(Asn1491Thrfs*10)	hom°	LPV	AR	*					
57	c.4470+1G>T, p.?	comp + known PV	PV	AR	*					
59	c.5118del, p.(Val1709Phefs*33)	hom	LPV	AR	*					
54	c.5340del, p.(Asp1781Metfs*6)	comp + c.3309del	LPV	AR	abs	−	−		+	−
***SPTLC1* (NM_006415)**										
63	c.397T>C, p.(Cys133Arg)	het	LPV	AD	*					
***WNK1* (NM_001184985)**										
73	c.2159del, p.(Pro720Argfs*35)	hom°	LPV	AR	red		−	+	+	−
74, 75	c.2392_2416del, p.(Ala798Profs*4)	hom	LPV	AR	red	−	−	+	+	−
76	c.2919_2920dup, p.(Pro974Hisfs*27)	hom°	LPV	AR	red	−	+	+	+	−
77	c.3071_3072del, p.(Asn1024Ilefs*28)	hom	LPV	AR	red		+	+		−
78	c.3909_3928del, p.(Gln1304Serfs*31)	comp + known PV	PV	AR	red				+	

abs = absent (i.e. complete pain loss), ACMG = American College of Medical Genetics; AD = autosomal dominant; AR = autosomal recessive; Au = autonomic dysfunction; comp = compound heterozygosity, confirmed by segregation analyses; F/M = fractures and/or mutilations; het = heterozygous; hom = homozygosity, confirmed by segregation analyses; hom° = homozygosity, but no parental samples available for segregation analyses; Inh. = inheritance; ID = intellectual disability; LPV = likely pathogenic variant; Mo = motor dysfunction; red = reduced; PP = pain perception; PV = pathogenic variant; SL = skin lesions (including ulcerations).

*Suspected clinical diagnosis of HSAN, no further clinical information available. For clinical data, ‘+’ indicates the presence and ‘–’ the absence of symptoms in the respective category.

**Table 2:** Novel variants in CIP/HSAN genes classified as likely pathogenic or VUS+ after reclassification

Patient	Novel variant	Genotype	ACMG	Reclass.	Inh.	PP	Au	Mo	SL	F/M	ID
***ATL3* (NM_015459)**											
1	c.544G>A, p.(Asp182Asn)	het	VUS	VUS+	AD	NP	+	+	+		−
2	c.1027A>G, p.(Met343Val)	het	VUS	VUS+	AD	red		−			
3	c.1053C>A, p.(Asn351Lys)	het	VUS	VUS+	AD	red		+	+	+	−
***DST* (NM_001374736)**											
5	c.19451A>T, p.(Gln6484Leu)	comp + c.22513C>T	VUS	LPV	AR	red	+	+	+	+	−
4	c.19942G>A, p.(Val6648Ile)	het + c.4849C>T (het)	VUS	VUS+	AR	red		−	+	+	−
***FLVCR1* (NM_014053)**											
7	c.655G>A, p.(Gly219Ser)	comp + c.868_871del	VUS	LPV	AR	abs		+	−	−	−
6	c.722C>T, p.(Ala241Val)	het + c.139_151del (het)	VUS	VUS+	AR	red	−	+	−	+	+
8	c.758T>A, p.(Phe253Tyr)	het + c.1369G>A (het)	VUS	LPV	AR	abs		+	+		
9	c.1034C>G, p.(Thr345Ser)	hom°	VUS	VUS+	AR	red		+	+	+	
10	c.1317G>A, p.(Met439Ile)	comp + c.1318_1321del	VUS	LPV	AR	red		+			−
8	c.1369G>A, p.(Glu457Lys)	het + c.758T>A (het)	VUS	VUS+	AR	abs		+	+		
11	c.1526-3C>T, p.?	comp + c.1194C>A	VUS	VUS+	AR	abs		−	+	+	−
***NTRK1* (NM_002529)**											
20, 21	c.287+5G>A, p.?	hom	VUS	VUS+	AR	abs	+		+	+	
25, 26	c.717+4A>T, p.?	comp + known PV/LPV	VUS	LPV	AR	*					
29	c.1136T>A, p.(Met379Lys)	hom	VUS	LPV	AR	red	+	+	+	+	+
31	c.1514T>A, p.(Ile505Asn)	hom	VUS	VUS+	AR	*					
***PRDM12* (NM_021619)**											
36	c.131_139del, p.(Val44_Gly46del)	hom	VUS	LPV	AR	red					+
***RAB7A* (NM_004637)**											
39, 40	c.467C>T, p.(Ala156Val)	het	VUS	VUS+	AD	red		+	−	+	−
***SCN9A* (NM_002977)**											
49	c.1650C>G, p.(Ser550Arg)	comp + c.1660C>A	VUS	VUS+	AR	*					
49	c.1660C>A, p.(Leu554Ile)	comp + c.1650C>G	VUS	VUS+	AR	*					
53	c.2689_2691del, p.(Trp897del)	hom°	VUS	LPV	AR	*					
58	c.5059G>C, p.(Ala1687Pro)	comp + known PV	VUS	LPV	AR	*					
***SPTLC1* (NM_006415)**											
64	c.1037C>T, p.(Ala346Val)	het	VUS	VUS+	AD	*					
***SPTLC2* (NM_004863)**											
65	c.302A>G, p.(His101Arg)	het	VUS	VUS+	AD	*					
66	c.359A>G, p.(Asn120Ser)	het	VUS	VUS+	AD	*					
67	c.430G>A, p.(Ala144Thr)	het	VUS	VUS+	AD	*					
68	c.707G>T, p.(Gly236Val)	het	VUS	VUS+	AD	*					
69, 70	c.1276A>T, p.(Ile426Phe)	het	VUS	VUS+	AD	NP	+	+	−	−	−
71	c.1304G>T, p.(Gly435Val)	het	VUS	VUS+	AD	*					
72	c.1513G>A, p.(Glu505Lys)	het	VUS	VUS+	AD	red		+	+	+	−

abs = absent (i.e. complete pain loss); AD = autosomal dominant; AR = autosomal recessive; Au = autonomic dysfunction; comp = compound heterozygosity, confirmed by segregation analyses; F/M = fractures and/or mutilations; het = heterozygous; hom = homozygosity, confirmed by segregation analyses; hom° = homozygosity, but no parental samples available for segregation analyses; Inh. = inheritance; ID = intellectual disability; LPV = likely pathogenic variant; Mo = motor dysfunction; NP = neuropathic pain; red = reduced; PP = pain perception; PV = pathogenic variant; Reclass. = reclassification; SL = skin lesions (including ulcerations); VUS = variant of uncertain significance.

*Suspected clinical diagnosis of HSAN, no further clinical information available. For clinical data, ‘+’ indicates the presence and ‘–’ the absence of symptoms in the respective category.

### Additional methods

Detailed information about long-read next-generation sequencing (NGS), sphingolipid profiling and electrophysiology is provided in the Supplementary material.

## Results

The cohort studied was composed of patients who presented at the participating centres or whose findings were referred from peripheral hospitals or treating physicians. The inclusion of patients from countries or regions with limited resources led to variable availability of clinical information (Supplementary Fig. 1). Inclusion criteria for the genetic test were the suspicion of CIP/HSAN due to a decreasing or absent pain sensation and written consent to participate in the study. Cases were excluded if a secondary cause of insensitivity to pain such as leprosy or abusive injury was confirmed or suspected. The predefined inclusion and exclusion criteria are detailed in the ‘Materials and methods’ section. A cohort of 78 patients from 73 families with the suspected diagnosis of CIP/HSAN had been analysed by NGS using gene panels, whole exome (WES) or whole genome sequencing (WGS). The core genes included in the analysis were the following 22 genes: *ATL1*, *ATL3*, *DST*, *ELP1*, *GLA*, *KIF1A*, *NGF*, *NTRK1*, *PRDM12*, *RAB7A*, *RETREG1*/*FAM134B*, *SCN9A*, *SCN11A*, *SPTLC1*, *SPTLC2*, *TTR*, *WNK1*, *MPV17*, *NAGLU*, *CLTCL1*, *FAAHP1* and *FLVCR1*.

**Figure 1:**
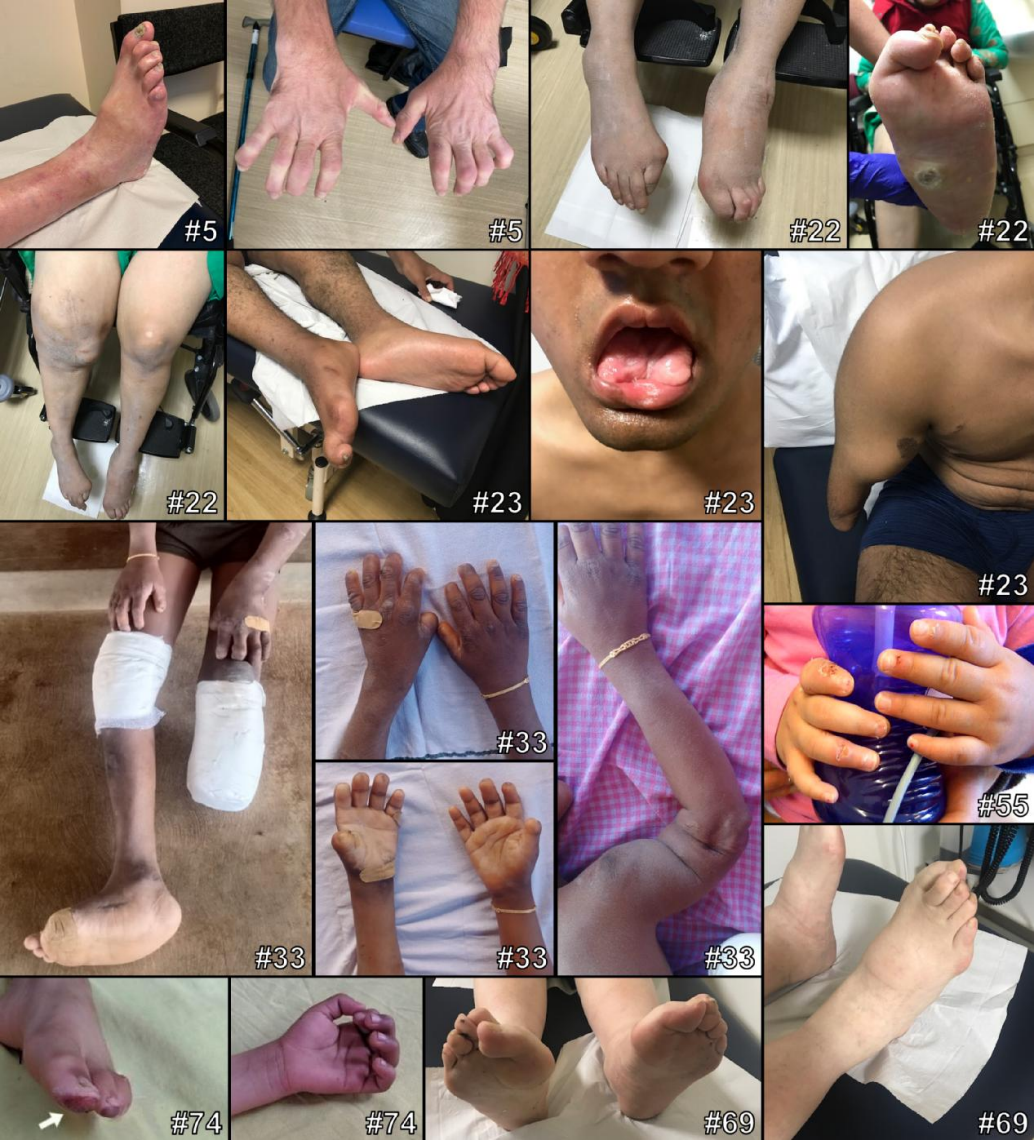
**Phenotypic findings in CIP/HSAN patients.** The pictures show exemplary findings in patients with novel (likely) pathogenic variants in *DST* (Patient 5), *NTRK1* (Patients 22, 23 and 33), *SCN9A* (Patient 55), *SPTLC2* (Patient 69), and *WNK1* (Patient 74). Additional clinical details are provided in Supplementary Table 1. Number sign indicates patient ID.

**Figure 2:**
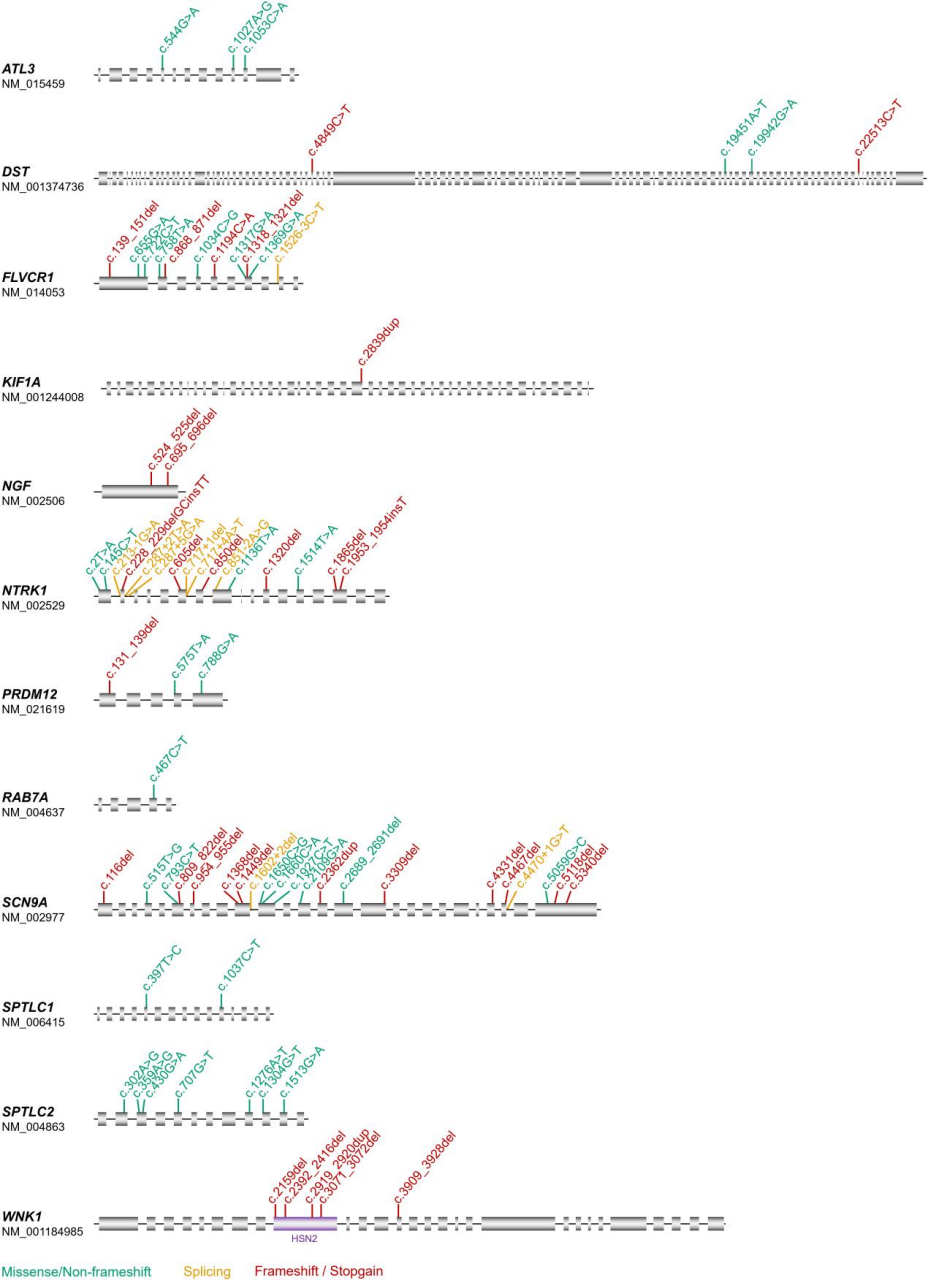
**Novel variants in known CIP/HSAN genes.** The location within the respective genes is shown for all variants identified in this study. Green = missense/non-frameshift; yellow = splicing; red = truncating. For *WNK1*, the neuron-specific exon HSN2 is highlighted, in which the majority of the known pathogenic variants (to date) is located.

By applying the ACMG criteria and our additional criteria for pathogenicity, 80 novel CIP/HSAN-related variants were identified in 78 patients (Tables 1 and 2 and Supplementary Table 3). Complete clinical and genetic details are given in Supplementary Table 1 and clinical images for a subset of patients are shown in Fig. 1. In some patients with recessive conditions, the novel variant occurred in compound heterozygosity with a second, previously described pathogenic or likely pathogenic variant as indicated in Tables 1 and 2. The mutation spectrum across all genes included missense variants, non-frameshift variants, frameshift variants and stop-gains whereby for some genes, exclusively one mutation type was found (e.g. missense variants in *SPTLC1* and *SPTLC2*) (Fig. 2). For most genes, no mutational hot spot was identified and the variants showed a distribution pattern across the entire coding regions, for some genes also including adjacent splice sites. An exception was the *WNK1* gene, for which a known clustering of variants was confirmed in the HSN2 exon known to code for part of the neuron-specific isoform. One of these variants, however, affected the pan-isoform of *WNK1* in compound heterozygosity with an HSN2-specific variant.

Novel CIP/HSAN-related variants were identified in 12 different CIP/HSAN genes, namely in *ATL3* (*n* = 3), *DST* (*n* = 2), *KIF1A* (*n* = 1), *NGF* (*n* = 2), *NTRK1* (*n* = 21), *PRDM12* (*n* = 3), *RAB7A* (*n* = 2), *SCN9A* (*n* = 22), *SPTLC1* (*n* = 2), *SPTLC2* (*n* = 8), *WNK1* (*n* = 6) and *FLVCR1* (*n* = 6) (Supplementary Fig. 2), where ‘*n*’ corresponds to the number of patients per gene.

Genomic data revealed a larger intragenic deletion of 1.3 kb (Patient 35) in *NTRK1* and two larger intragenic deletions of 3.8 kb (Patient 60) and 3.4 kb (Patient 61) in *SCN9A*, respectively. Patient 62 showed a 322 kb deletion spanning the entire *SCN9A* locus. In these patients, the deletion was in a compound heterozygous situation with a single nucleotide variant on the other allele. For the 3.4 kb deletion (Patient 61) spanning exon 20 of *SCN9A*, the DNA quality was sufficient to determine the exact size of the maternally inherited deletion by long-read sequencing (Oxford Nanopore Technologies, ONT) (Supplementary Fig. 3A). The deletion had a size of 3421 bp [chr2:166,235,764-166,239,185 (hg38)] and was further confirmed by quantitative PCR (qPCR) (Supplementary Fig. 3B). The father is a carrier of the single base pair deletion c.5318del, confirming compound heterozygosity in the index patient. For eight patients carrying *SPTLC1* and *SPTLC2* variants, plasma samples were available and 1-deoxy-sphingolipids (1-deoxySL) levels were elevated in line with assumed pathogenicity of the variant (Patients 63–68 and Patients 71–72) (Supplementary Fig. 4). For the homozygous missense variant p.(Leu172Arg) in Na_v_1.7 (*SCN9A*) in transmembrane segment 2 of channel domain I (D1), *in silico* predictions regarding a role in CIP were inconsistent, so functional studies were performed (Patient 42). The respective variant was electrophysiologically analysed upon heterologous expression in HEK293 cells and showed a complete loss-of-function in line with pathogenicity (Supplementary Fig. 5).

## Discussion

This retrospective cross-sectional study was aimed at molecular characterization of patients with CIP/HSAN and to our knowledge, includes the largest number of molecularly resolved cases to date. The study has its main limitation in that in some cases the inclusion criteria were met, but detailed clinical data were not available. This was because data were collected from multiple sites, some with only limited clinical research and documentation capabilities. This is a frequently observed difficulty in ultrarare diseases, as access to patients is already a major hurdle. In addition, the total number of cases investigated at each centre over the years is variable, making it difficult to accurately determine detection rates for CIP/HSAN. The strength and focus of this study were therefore on molecular characterization and careful assessment of the pathogenicity of variants in CIP/HSAN-associated genes. Thus, a large cohort of patients with these extremely rare diseases could be studied in a collaborative network and patients from normally under-represented countries could also be included.

Overall, the study supports that *SCN9A* and *NTRK1* are the most frequently mutated genes in congenital painlessness. Whereas CIP/HSAN-related pathogenic *SCN9A* variants frequently lead to anosmia as a secondary symptom, *NTRK1*-related neuropathy is accompanied by lack of sweat gland innervation with anhidrosis and sometimes life-threatening hyperthermia. Intellectual disability was not observed in *SCN9A*-associated neuropathy and was present with a variable degree in *NTRK1*-related disease. In adult onset HSN/HSAN, pathogenic variants are most frequently found in the enzymes of the sphingolipid metabolism pathway (*SPTLC1/2*). The study has a bias at this point: previously, more pathogenic variants were described for *SPTLC1* than for *SPTLC2*. We report more cases with *SPTLC2* variants in this study, but this is due to the fact that we solely report new disease-relevant variants. In the UK, for example, there is a high frequency of *SPTLC1*-related patients due to the p.(Cys133Trp) founder mutation.

In addition, to address these more frequently mutated genes, we were able to substantiate the role of genes that have so far only very rarely been described as the cause of CIP/HSAN. To date, only three causal variants have been reported in *NGF*, two of which are missense variants.^10,20,21^ The study expands the mutation spectrum to include two homozygous loss-of-function variants and further corroborates a clinical presentation broadly equivalent to that of pathogenic variants in the NGF receptor encoding gene, *NTRK1*.

For *ATL3*, only two causal missense variants have been described to date.^6,22^ As for the previous changes, the here identified heterozygous variants are missense changes located at very highly conserved residues of the protein. The data further support that a dominant-negative effect of missense variants is likely the central mechanism of *ATL3*-associated disease. A recent report of an early stop-gain variant in *ATL3*, p.(Arg6Ter)^23^ would argue against this assumption, but proof of pathogenicity of this variant is pending. *FLVCR1* variants have predominantly been reported in cases of autosomal-recessive posterior column ataxia with retinitis pigmentosa (PCARP).^24^ Since the first description of *FLVCR1* variants as cause of HSAN, only a few pathogenic variants have been reported, including missense and loss-of-function variants.^25–27^ The findings in this study based on six additional patients show that the clinical transitions between PCARP and HSAN are fluid and often result in a complex phenotype with overlapping symptoms.

In several patients, the underlying genetic variants are immediately classifiable as likely pathogenic or pathogenic according to ACMG criteria. A greater difficulty arises with missense variants that often have to be classified as VUS. Here, further parameters such as homozygosity/compound heterozygosity with a likely pathogenic or very rare variant for recessive disorders, occurrence in more than one affected individual, a highly specific phenotype, detailed evaluation of the functionally critical domains and amino acids of the protein, or the lack of evidence of another genetic cause by broad genetic screening using WES or WGS were used to prove the pathogenicity of the suspicious variants. For all variants listed in this study, those that were formally classified as VUS according to ACMG criteria were considered as VUS+ (i.e. assumed to be likely pathogenic) or likely pathogenic based on such additional criteria. This critical review of variants was performed to exclude false positives as much as possible and to provide reliable genetic counselling to patients and their families.

Further functional assessment of VUS in *SPTLC1* or *SPTLC2* was achieved by detecting toxic sphingolipid species in the serum of patients by mass spectrometry. Both genes encode key enzymes of the *de novo* sphingolipid synthesis pathway, the so-called serine palmitoyltransferases (SPT). Pathogenic gain-of-function variants in *SPTLC1* and *SPTLC2* lead to the increased formation of toxic 1-deoxySL, which have been measured in patient’s plasma to further assess suspicious VUS. Heterologous expression studies with functional measures were additionally performed in selected cases to corroborate pathogenicity of a VUS. As an additional example, we show whole-cell voltage-clamp recordings of HEK293 cells transiently expressing the *SCN9A* missense variant p.(Leu172Arg), confirming a complete loss-of-function of this variant.

The analysis of larger deletions has long been a difficulty in NGS-based diagnostics, but with increasingly better bioinformatic algorithms and new sequencing methods, this type of genetic alteration can more frequently be detected in CIP/HSAN. In rare cases, large deletions in *NTRK1* have already been reported in HSAN.^28^ Our results show another case of an *NTRK1* deletion and, in addition, three patients with larger deletions in *SCN9A* confirming their expected relevance in CIP/HSAN. Determination of copy number variants (CNV) from genomic data is therefore generally recommended in the case of an underlying loss-of-function mechanism. Long-read sequencing technologies, such as nanopore sequencing, have proven useful for rapidly determining the size and position of deletions with base pair precision,^29^ as we also exemplify in a case of a 3.4 kb *SCN9A* deletion.

Another feature in CIP/HSAN concerns isoform-specific pathogenic variants. HSAN-relevant recessive variants in *WNK1* cluster in a neuron-specific alternatively spliced exon (HSN2 exon) of the gene, whereas biallelic pan-*WNK1* loss is most likely lethal. We report here one of the rare cases of a compound heterozygosity for a mutation in the neuron-specific exon in trans with a loss-of-function mutation affecting the pan-isoform of *WNK1*, similarly to a previous report.^30^

In conclusion, our results broaden the mutational spectrum of CIP/HSAN and the cohort provides a framework for natural history studies and improvement of care in these rare debilitating conditions.

## Supplementary Material

awad328_Supplementary_Data

## Data Availability

The data that support the findings of this study are available from the corresponding author on reasonable request.

## References

[1] Cox JJ , Reimann F, Nicholas AK, et al An SCN9A channelopathy causes congenital inability to experience pain. Nature. 2006;444:894–898. 17167479 10.1038/nature05413 PMC7212082

[2] Leipold E , Liebmann L, Korenke GC, et al A de novo gain-of-function mutation in SCN11A causes loss of pain perception. Nat Genet. 2013;45:1399–1404. 24036948 10.1038/ng.2767

[3] Dawkins JL , Hulme DJ, Brahmbhatt SB, Auer-Grumbach M, Nicholson GA. Mutations in SPTLC1, encoding serine palmitoyltransferase, long chain base subunit-1, cause hereditary sensory neuropathy type I. Nat Genet. 2001;27:309–312. 11242114 10.1038/85879

[4] Rotthier A , Auer-Grumbach M, Janssens K, et al Mutations in the SPTLC2 subunit of serine palmitoyltransferase cause hereditary sensory and autonomic neuropathy type I. Am J Hum Genet. 2010;87:513–522. 20920666 10.1016/j.ajhg.2010.09.010 PMC2948807

[5] Guelly C , Zhu PP, Leonardis L, et al Targeted high-throughput sequencing identifies mutations in atlastin-1 as a cause of hereditary sensory neuropathy type I. Am J Hum Genet. 2011;88:99–105. 21194679 10.1016/j.ajhg.2010.12.003 PMC3014370

[6] Kornak U , Mademan I, Schinke M, et al Sensory neuropathy with bone destruction due to a mutation in the membrane-shaping atlastin GTPase 3. Brain. 2014;137(Pt 3):683–692. 24459106 10.1093/brain/awt357

[7] Kurth I , Pamminger T, Hennings JC, et al Mutations in FAM134B, encoding a newly identified Golgi protein, cause severe sensory and autonomic neuropathy. Nat Genet. 2009;41:1179–1181. 19838196 10.1038/ng.464

[8] Rivière JB , Ramalingam S, Lavastre V, et al KIF1A, An axonal transporter of synaptic vesicles, is mutated in hereditary sensory and autonomic neuropathy type 2. Am J Hum Genet. 2011;89:219–230. 21820098 10.1016/j.ajhg.2011.06.013 PMC3155159

[9] Verhoeven K , De Jonghe P, Coen K, et al Mutations in the small GTP-ase late endosomal protein RAB7 cause Charcot-Marie-Tooth type 2B neuropathy. Am J Hum Genet. 2003;72:722–727. 12545426 10.1086/367847 PMC1180247

[10] Einarsdottir E , Carlsson A, Minde J, et al A mutation in the nerve growth factor beta gene (NGFB) causes loss of pain perception. Hum Mol Genet. 2004;13:799–805. 14976160 10.1093/hmg/ddh096

[11] Indo Y , Tsuruta M, Hayashida Y, et al Mutations in the TRKA/NGF receptor gene in patients with congenital insensitivity to pain with anhidrosis. Nat Genet. 1996;13:485–488. 8696348 10.1038/ng0896-485

[12] Baets J , Duan X, Wu Y, et al Defects of mutant DNMT1 are linked to a spectrum of neurological disorders. Brain. 2015;138(Pt 4):845–861. 25678562 10.1093/brain/awv010 PMC5014076

[13] Edvardson S , Cinnamon Y, Jalas C, et al Hereditary sensory autonomic neuropathy caused by a mutation in dystonin. Ann Neurol. 2012;71:569–572. 22522446 10.1002/ana.23524

[14] Palma JA , Yadav R, Gao D, Norcliffe-Kaufmann L, Slaugenhaupt S, Kaufmann H. Expanding the genotypic spectrum of congenital sensory and autonomic neuropathies using whole-exome sequencing. Neurol Genet. 2021;7:e568. 33884296 10.1212/NXG.0000000000000568 PMC8054964

[15] Yuan JH , Higuchi Y, Ando M, et al Multi-type RFC1 repeat expansions as the most common cause of hereditary sensory and autonomic neuropathy. Front Neurol. 2022;13:986504. 36061987 10.3389/fneur.2022.986504 PMC9428154

[16] Garofalo K , Penno A, Schmidt BP, et al Oral L-serine supplementation reduces production of neurotoxic deoxysphingolipids in mice and humans with hereditary sensory autonomic neuropathy type 1. J Clin Invest. 2011;121:4735–4745. 22045570 10.1172/JCI57549 PMC3225995

[17] Fridman V , Suriyanarayanan S, Novak P, et al Randomized trial of l-serine in patients with hereditary sensory and autonomic neuropathy type 1. Neurology. 2019;92:e359–e370. 30626650 10.1212/WNL.0000000000006811 PMC6345118

[18] Auranen M , Toppila J, Suriyanarayanan S, et al Clinical and metabolic consequences of L-serine supplementation in hereditary sensory and autonomic neuropathy type 1C. Cold Spring Harb Mol Case Stud. 2017;3:a002212. 29042446 10.1101/mcs.a002212 PMC5701299

[19] Richards S , Aziz N, Bale S, et al Standards and guidelines for the interpretation of sequence variants: A joint consensus recommendation of the American college of medical genetics and genomics and the association for molecular pathology. Genet Med. 2015;17:405–424. 25741868 10.1038/gim.2015.30 PMC4544753

[20] Carvalho OP , Thornton GK, Hertecant J, et al A novel NGF mutation clarifies the molecular mechanism and extends the phenotypic spectrum of the HSAN5 neuropathy. J Med Genet. 2011;48:131–135. 20978020 10.1136/jmg.2010.081455 PMC3030776

[21] Shaikh SS , Nahorski MS, Woods CG. A third HSAN5 mutation disrupts the nerve growth factor furin cleavage site. Mol Pain. 2018;14:1744806918809223. 30296891 10.1177/1744806918809223 PMC6207963

[22] Fischer D , Schabhuttl M, Wieland T, Windhager R, Strom TM, Auer-Grumbach M. A novel missense mutation confirms ATL3 as a gene for hereditary sensory neuropathy type 1. Brain. 2014;137(Pt 7):e286. 24736309 10.1093/brain/awu091

[23] Mohammadi S , Jafari Khamirani H, Baneshi M, et al A novel nonsense variant in the ATL3 gene is associated with disturbed pain sensitivity, numbness of distal limbs and muscle weakness. Ann Hum Genet. 2023;87:147–157. 36856139 10.1111/ahg.12501

[24] Rajadhyaksha AM , Elemento O, Puffenberger EG, et al Mutations in FLVCR1 cause posterior column ataxia and retinitis pigmentosa. Am J Hum Genet. 2010;87:643–654. 21070897 10.1016/j.ajhg.2010.10.013 PMC2978959

[25] Chiabrando D , Castori M, di Rocco M, et al Mutations in the heme exporter FLVCR1 cause sensory neurodegeneration with loss of pain perception. PLoS Genet. 2016;12:e1006461. 27923065 10.1371/journal.pgen.1006461 PMC5140052

[26] Castori M , Morlino S, Ungelenk M, et al Posterior column ataxia with retinitis pigmentosa coexisting with sensory-autonomic neuropathy and leukemia due to the homozygous p.Pro221Ser FLVCR1 mutation. Am J Med Genet B Neuropsychiatr Genet. 2017;174:732–739. 28766925 10.1002/ajmg.b.32570

[27] Bertino F , Firestone K, Bellacchio E, et al Heme and sensory neuropathy: Insights from novel mutations in the heme exporter feline leukemia virus subgroup C receptor 1. Pain. 2019;160:2766–2775. 31408049 10.1097/j.pain.0000000000001675

[28] Li L , Jia C, Tang Y, Kong Y, Xia Y, Ma L. Novel gross deletion mutations in NTRK1 gene associated with congenital insensitivity to pain with anhidrosis. Front Pediatr. 2021;9:638190. 33748046 10.3389/fped.2021.638190 PMC7969531

[29] Kraft F , Kurth I. Long-read sequencing to understand genome biology and cell function. Int J Biochem Cell Biol. 2020;126:105799. 32629027 10.1016/j.biocel.2020.105799

[30] Shekarabi M , Girard N, Riviere JB, et al Mutations in the nervous system–specific HSN2 exon of WNK1 cause hereditary sensory neuropathy type II. J Clin Invest. 2008;118:2496–2505. 18521183 10.1172/JCI34088 PMC2398735

