# Supplementary material for: Genetic landscape of congenital insensitivity to pain and hereditary sensory and autonomic neuropathies
Source: Brain. 2023 Sep 28;146(12):4880–90. doi: 10.1093/brain/awad328 (PMC10689924; doi:10.1093/brain/awad328)
Supplement: awad328_Supplementary_Data [file awad328_supplementary_data.zip › brain-2023-00799-File007.pdf]

Supplementary Material - Methods

Long-read next-generation sequencing

Whole genome long-read sequencing was done using Oxford Nanopore Technologies (ONT) in patient 61 to confirm the presence of the large deletion in *SCN9A* detected by WES and to determine its exact size. Briefly, 10 µg of DNA was sheared in 100 µL H_2O by pulling it ten times through a HPLC injection needle (blunt metal needles [91029] Hamilton, attached to a 1 mL BD Luer Lock syringe) and cleaned up with 0.4X AMPure XP beads. Approximately 3.5–5 µg of DNA was used for the library preparation with the 1D Ligation Sequencing Kit (SQK-LSK108) and sequenced on the GridION sequencer (ONT) utilizing R9.4.1 flow cells. Base calling was done using guppy (ONT), adapters were trimmed by porechop and reads were filtered with NanoFilt[1] for quality >8. Alignment was done by minimap2[2] and bam files were generated using samtools.[3] Structural variants were called using NanoSV[4] and picky.[5] Tandem repeat lengths within the extracted reads were further analysed by NanoSatellite.[6]

Sphingolipid profiles

Plasma sphingolipid profiles were measured in patients with *SPTLC1* or *SPTLC2* variants. The extracted plasma sphingolipids were subjected to an acid/base hydrolysis to release the free sphingoid bases from the conjugated N-acyl chains and headgroups. The profiling included C16SO, C16SA, C17SO, C17SA, C18SO, C18SA, C19SO, C20SO, C20SA, sphingadiene, 1-deoxysphingosine (1-deoxySO), and 1-deoxy-sphinganine (1- deoxySA). Details on the procedure have been described earlier.[7,8]

Electrophysiology

Mutation p.(Leu172Arg) was introduced into a plasmid encoding the human $Na_V1.7$ (UniProt Q15858) channel using a PCR-based strategy and the coding sequence was verified by sanger sequencing. Human embryonic kidney 293 cells (HEK293 cells; CAMR, Porton Down, Salisbury, UK) were maintained and transfected with constructs encoding $Na_V1.7$ or $Na_V1.7$-L172R according to a previously described procedure.[9] HEK293 cells transfected with a plasmid lacking the $Na_V1.7$ coding sequence served as controls.

Whole-cell Na^+ current densities were recorded 24–48 h after transfection using an EPC-10 patch-clamp amplifier operated by PatchMaster software (both HEKA Elektronik, Lambrecht, Germany). Patch pipettes were pulled from borosilicate glass, coated with silicon elastomer to reduce tip capacitance, and series resistance was corrected electronically up to 85%. All experiments were performed at 20 ± 1 °C. Patch pipettes were filled with (in mM): 35 NaCl, 105 CsF, 10 EGTA, 10 HEPES (pH 7.4 with CsOH); the bath solution contained (in mM): 150 NaCl, 2 KCl, 1.5 $CaCl_2$, 1 $MgCl_2$, 10 HEPES (pH 7.4 with NaOH). Activation of Na_V channels was triggered with 20-ms depolarizing test pulses ranging from –120 to 65 mV, applied every 5 s in steps of 5 mV. Steady-state channel inactivation was measured with a test pulse to –20 mV applied after a 500-ms conditioning period in which the membrane voltage was varied in 10 mV steps between –140 and 0 mV. Holding potential was –120 mV throughout. All data are presented as mean peak current densities ± SEM (*n*), where *n* is the number of independent experiments.

References

1. De Coster W, D'Hert S, Schultz DT, Cruts M, Van Broeckhoven C. NanoPack: visualizing and processing long-read sequencing data. *Bioinformatics*. Aug 1 2018;34(15):2666-2669. doi:10.1093/bioinformatics/bty149

2. Li H. Minimap2: pairwise alignment for nucleotide sequences. *Bioinformatics*. Sep 15 2018;34(18):3094-3100. doi:10.1093/bioinformatics/bty191

3. Li H, Handsaker B, Wysoker A, et al. The Sequence Alignment/Map format and SAMtools. *Bioinformatics*. Aug 15 2009;25(16):2078-9. doi:10.1093/bioinformatics/btp352

4. Cretu Stancu M, van Roosmalen MJ, Renkens I, et al. Mapping and phasing of structural variation in patient genomes using nanopore sequencing. *Nat Commun*. Nov 6 2017;8(1):1326. doi:10.1038/s41467-017-01343-4

5. Gong L, Wong CH, Cheng WC, et al. Picky comprehensively detects high-resolution structural variants in nanopore long reads. *Nat Methods*. Jun 2018;15(6):455-460. doi:10.1038/s41592-018-0002-6

6. De Roeck A, De Coster W, Bossaerts L, et al. NanoSatellite: accurate characterization of expanded tandem repeat length and sequence through whole genome long-read sequencing on PromethION. *Genome Biol*. Nov 14 2019;20(1):239. doi:10.1186/s13059-019-1856-3

7. Mwinyi J, Bostrom A, Fehrer I, et al. Plasma 1-deoxysphingolipids are early predictors of incident type 2 diabetes mellitus. *PLoS One*. 2017;12(5):e0175776. doi:10.1371/journal.pone.0175776

8. Penno A, Reilly MM, Houlden H, et al. Hereditary sensory neuropathy type 1 is caused by the accumulation of two neurotoxic sphingolipids. *J Biol Chem*. Apr 9 2010;285(15):11178-87. doi:10.1074/jbc.M109.092973

9. Leipold E, Markgraf R, Miloslavina A, et al. Molecular determinants for the subtype specificity of mu-conotoxin SIIIA targeting neuronal voltage-gated sodium channels. *Neuropharmacology*. Jul-Aug 2011;61(1-2):105-11. doi:10.1016/j.neuropharm.2011.03.008

Supplementary Material – Tables & Figures

Supplementary Table 2. Modified classification criteria for the interpretation of sequence variants. Original ACMG criteria are shown in black, modified criteria are shown in red.

**Very strong:**	
**PVS1**	Null variant (nonsense, frameshift, canonical ±1 or 2 splice sites, initiation codon, single or multiexon deletion) in a gene where LOF is a known mechanism of disease
**Strong:**	
**PS1**	Same amino acid change as a previously established pathogenic variant regardless of nucleotide change
**PS2**	De novo (both maternity and paternity confirmed) in a patient with the disease and no family history
**PS3**	Well-established in vitro or in vivo functional studies supportive of a damaging effect on the gene or gene product
**PS4**	The prevalence of the variant in affected individuals is significantly increased compared with the prevalence in controls
**Moderate:**	
**PM1**	Located in a mutational hot spot and/or critical and well-established functional domain (e.g., active site of an enzyme) without benign variation
**PM2**	Absent from controls (or at extremely low frequency if recessive) in Exome Sequencing Project, 1000 Genomes Project, or Exome Aggregation Consortium
**PM3**	For recessive disorders, detected in trans with a pathogenic variant
**PM4**	Protein length changes as a result of in-frame deletions/insertions in a nonrepeat region or stop-loss variants
**PM5**	Novel missense change at an amino acid residue where a different missense change determined to be pathogenic has been seen before
**PM6**	Assumed de novo, but without confirmation of paternity and maternity
**mPM7**	For recessive disorders, detected in trans with a **likely** pathogenic variant
**mPM8**	Observation of the variant in multiple unrelated patients with the same phenotype, while absent from controls
**Supporting:**	
**PP1**	Cosegregation with disease in multiple affected family members in a gene definitively known to cause the disease
**PP2**	Missense variant in a gene that has a low rate of benign missense variation and in which missense variants are a common mechanism of disease
**PP3**	Multiple lines of computational evidence support a deleterious effect on the gene or gene product (conservation, evolutionary, splicing impact, etc.)
**PP4**	Patient's phenotype or family history is highly specific for a disease with a single genetic etiology
**PP5**	Reputable source recently reports variant as pathogenic, but the evidence is not available to the laboratory to perform an independent evaluation
**mPP6**	For recessive disorders, detected in homozygous state or in trans with a second extremely rare variant
**mPP7**	Variant in a gene to which objectively measurable parameters in the patient point specifically, due to well-established knowledge of metabolic processes
**mPP8**	Other possible genetic causes have been excluded by broad screening of phenotype-related genes (whole exome or genome) and common non-genetic causes have been excluded to the best possible extent

Supplementary Table 3. Large deletions in *NTRK1* and *SCN9A* classified as pathogenic.

Patient	Novel variant	Genotype	ACMG	Inh.	PP	Au	Mo	SL	F/M	ID
***NTRK1* (NM_002529)**										
35	1.3 kb deletion (exons 5 and 6)	comp+known PV	PV	AR	*					
***SCN9A* (NM_002977)**										
60	3.8 kb deletion (exon 8 and part of exon 9)	comp+known PV	PV	AR	*					
61	3.4 kb deletion (exon 20)	comp+known PV	PV	AR	abs	-		+	+	-
62	322 kb deletion of 2q24.3 (entire gene)	comp+known PV	PV	AR	*					

abs=Absent (=Complete pain loss), AR=Autosomal recessive, Au=Autonomic dysfunction, comp=Compound heterozygosity, confirmed by segregation analyses, F/M=Fractures and/or mutilations, Inh.=Inheritance, ID=Intellectual disability, Mo=Motor dysfunction, PP=Pain perception, PV=Pathogenic variant, SL=Skin lesions (including ulcerations). *Suspected clinical diagnosis of HSAN, no further clinical information available. For clinical data, "+" indicates the presence and "–" the absence of symptoms in the respective category.

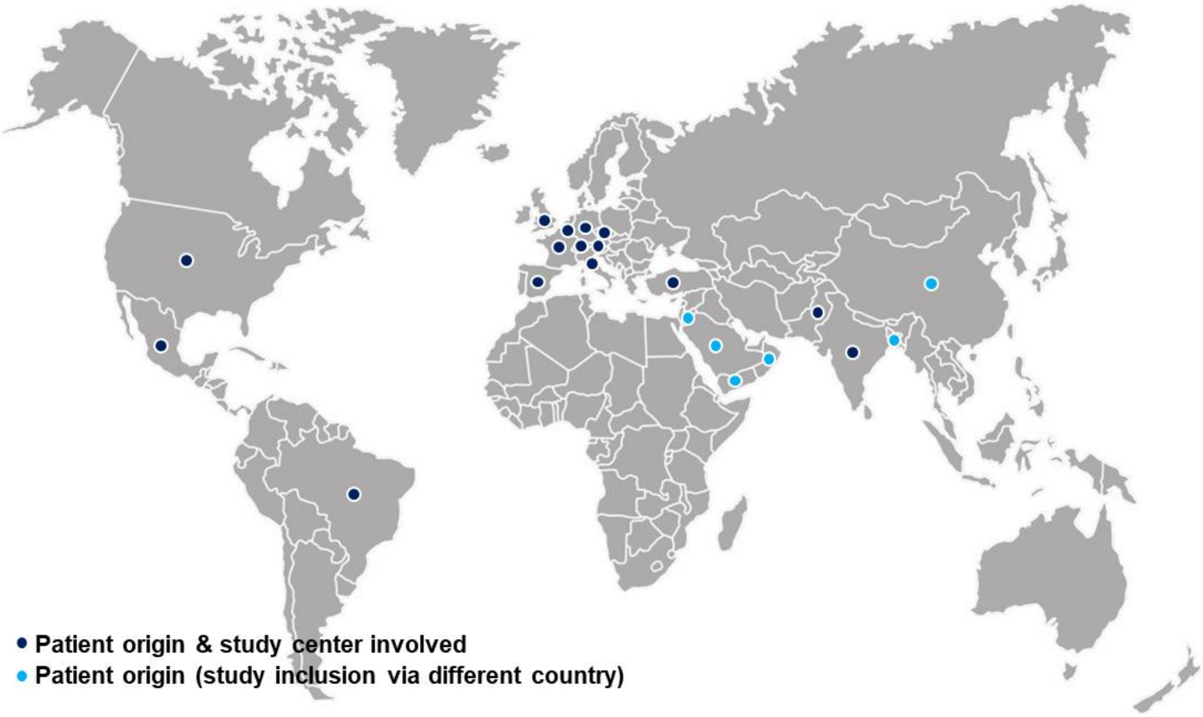

Supplementary Fig. 1: Origin of patients and study centers involved. Spots indicate the respective country, not city or region. Since the patients' origin was not available for all cases, no quantitative statements can be made. If multiple study centers and/or patients from the same country were involved, they were therefore summarized in one spot for the sake of simplicity.

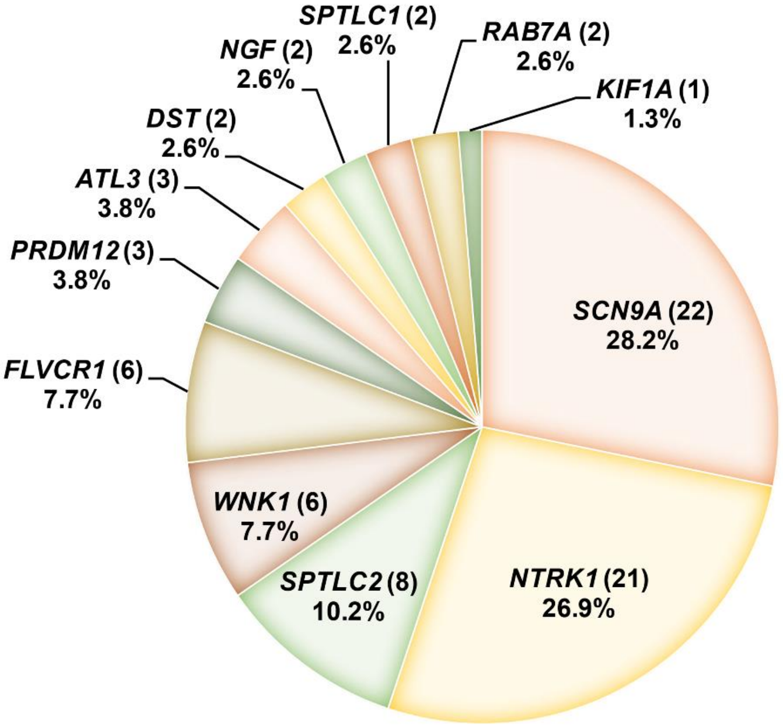

Supplementary Fig. 2: Distribution of the patients. Pie chart with the percentage distribution, the number of patients is shown in parentheses.

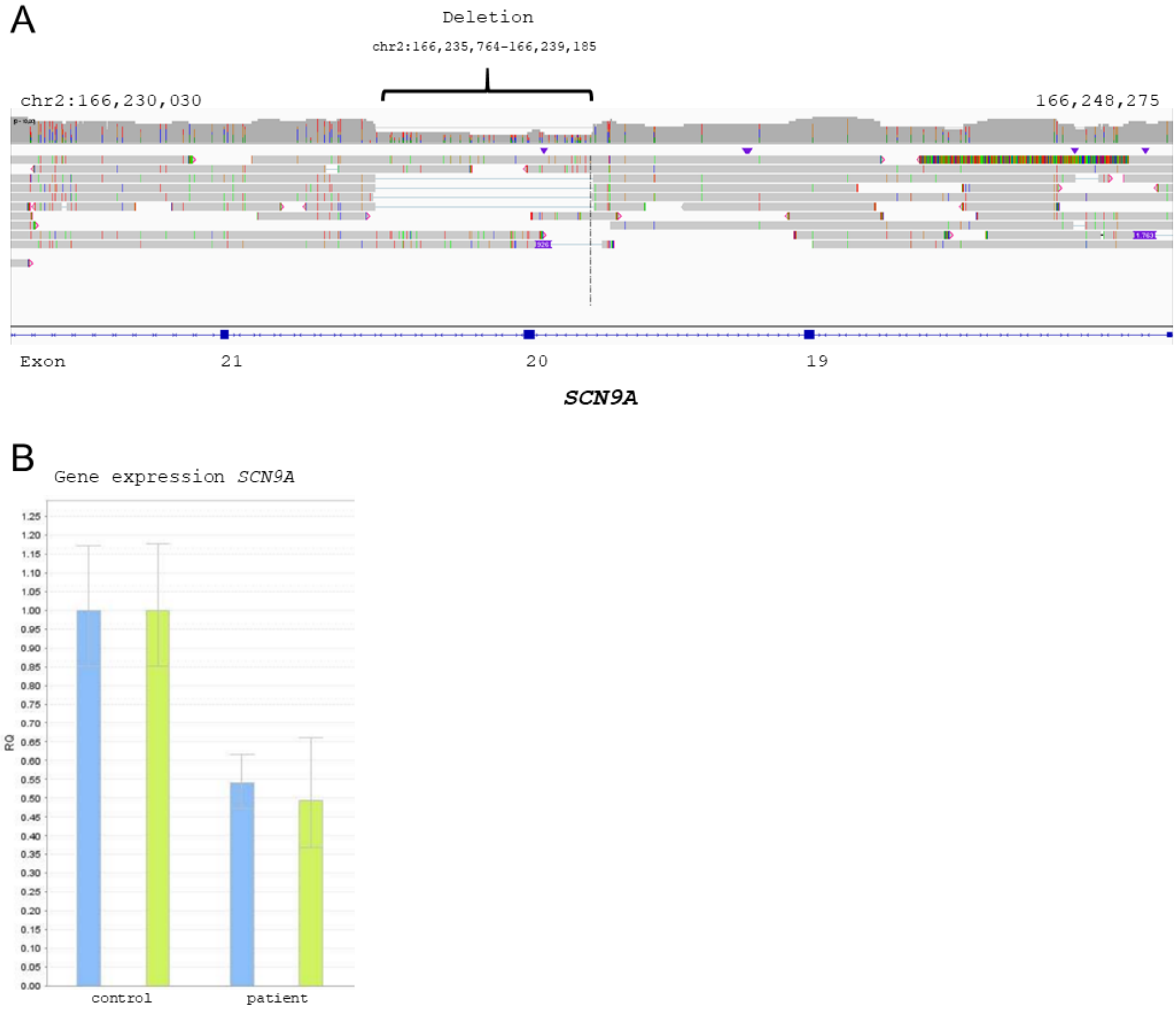

Supplementary Fig. 3: 3.4kb deletion in patient 61, spanning exon 20 of *SCN9A*. (A) Nanopore sequencing and **(B)** qPCR.

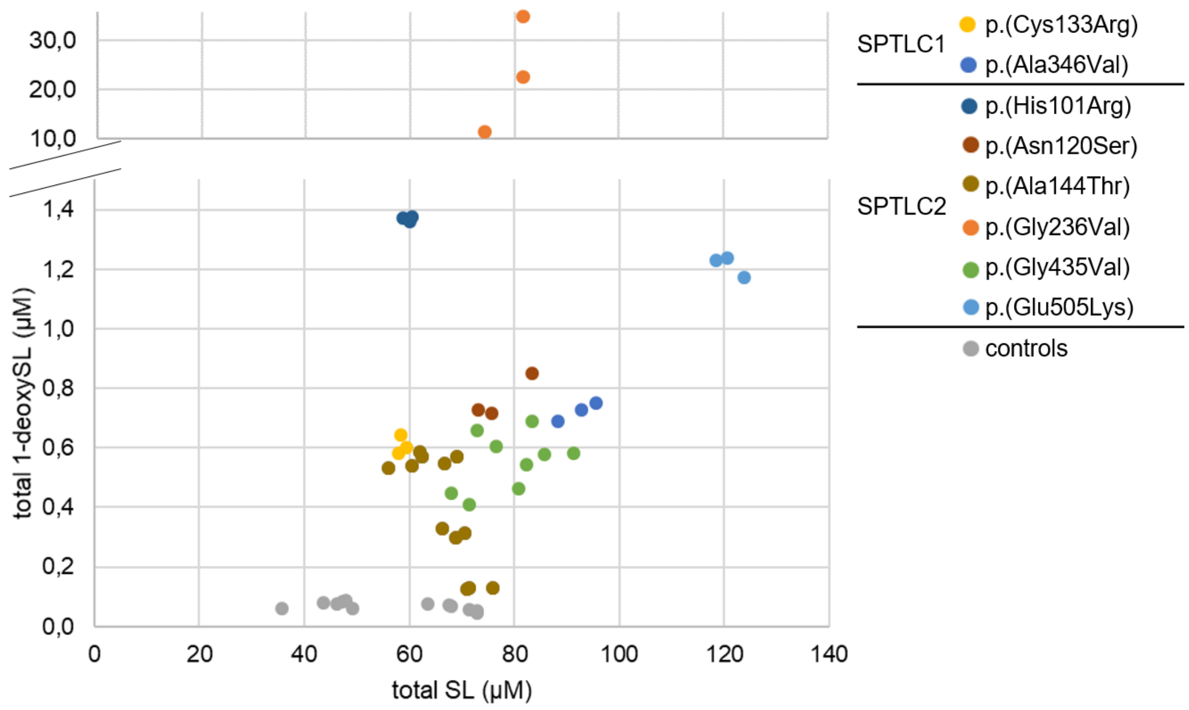

Supplementary Fig. 4: Elevated 1-deoxySL levels in plasma samples of HSAN patients.

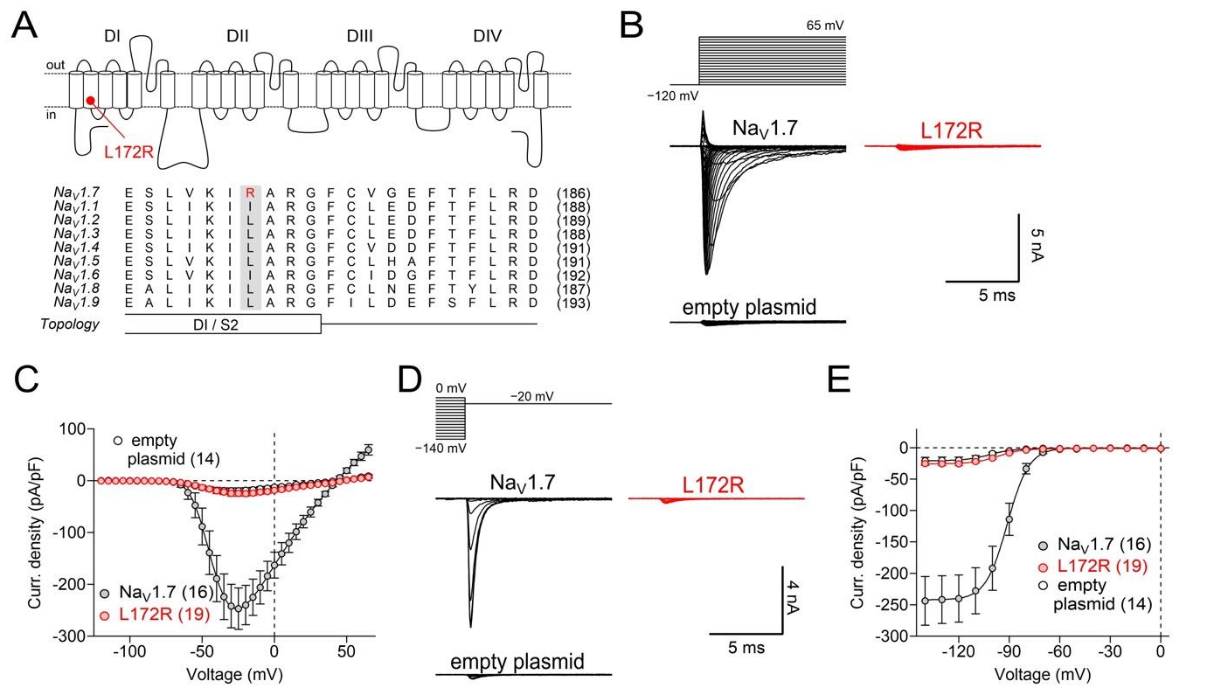

Supplementary Fig. 5: Variant p.(Leu172Arg) = p.(L172R) causes loss-of-function of Na$_V$1.7 channels. (**A**) Topological model (*top*) of the four-domain (DI–DIV) structure of the α-subunit of human Na$_V$1.7 highlighting the position of mutation p.L172R in transmembrane segment S2 in DI. *Bottom:* Amino acid sequence alignment of the nine human Na$_V$ isoforms (Na$_V$1.1–1.9) covering the C-terminal portion of DI/S2 and the intracellular DI/S2-S3. The arginine replacing leucine in position 172 in Na$_V$1.7 mutant channels is shown red. Numbers in parenthesis refer to the positions of the last amino acid residue shown with respect to the full-length protein sequences. (**B**) Representative whole-cell current traces recorded from HEK293 cells transfected with plasmids encoding Na$_V$1.7 (black, filled circles) or Na$_V$1.7-L172R (red) or with an empty control plasmid (black, open circles), in response to depolarizations ranging from –120 mV to 65 mV in steps of 5 mV. The corresponding pulse protocol is shown on top. (**C**) Peak current densities obtained from experiments as shown in *B*, plotted as a function of voltage. Superimposed curves are data fits describing the voltage-dependence of channel activation. (**D**) Representative whole-cell current traces recorded from HEK293 cells transfected with plasmids encoding Na$_V$1.7 (black, filled circles) or Na$_V$1.7-L172R (red) or with an empty control plasmid (black, open circles), recorded at –20 mV following a 500-ms conditioning period ranging from –140 to 0 mV in steps of 10 mV. The corresponding pulse protocol is shown on top. (**E**) Voltage-dependence of peak current densities characterizing steady-state channel inactivation, obtained from current responses as shown in *D*. Data points in *C*, *E* represent means ± SEM with numbers of experimental replicates, *n*, provided in parentheses.
